# Expression of the gene encoding oxalate decarboxylase from *Bacillus subtilis* and characterization of the recombinant enzyme

**DOI:** 10.1186/1756-0500-7-598

**Published:** 2014-09-03

**Authors:** Eunhye Lee, Byong Chang Jeong, Yong Hyun Park, Hyeon Hoe Kim

**Affiliations:** Clinical Research Institute, Seoul National University Hospital, Seoul, Korea; Department of Urology, Samsung Medical Center, Sungkyunkwan University School of Medicine, Seoul, Korea; Department of Urology, The Catholic University of Korea, Mary’s Hospital, Seoul St, Seoul Korea; Department of Urology, Seoul National University College of Medicine and Clinical Research Institute, 28 Yeongeon-dong, Jongno-gu, Seoul 110-744 Korea

**Keywords:** Oxalate, *Yvrk*, *Bacillus subtilis*

## Abstract

**Background:**

The concentration of urinary oxalate is more influential to the formation of calcium oxalate urolithiasis than is urinary calcium concentration. *YvrK* gene encodes a 43 KD-sized oxalate decarboxylase. We previously developed the recombinant *Escherichia coli (E. coli*) expressing *Yvrk* gene from *Bacillus subtilis* and named it as *pBy*. The aim of this study was to purify the recombinant oxalate decarboxylase overexpressed in *E. coli* and evaluate the oxalate-degrading activity of the purified enzyme.

**Results:**

The oxalate-degrading activity of *pBy* was highest when cultured at pH 5. The activity of purified oxalate decarboxylase was determined after incubation with sodium oxalate and the optimal pH and temperature of oxalate decarboxylase were determined. Purified oxalate decarboxylase degraded more than 50% of oxalate when incubated with MnCl_2_ and sodium oxalate in atmospheric O_2_. The optimal pH of recombinant oxalate decarboxylase was 5 and the optimal temperature was 28°C. Eight-week-old Sprague–Dawley male rats were used as a transient hyperoxaluric rat model. Suprapubic catheter was inserted into the bladder of each rat and urine was collected hourly before and 3 hours after oral oxalate intake in the absence and presence of homogenates of *pBy* and non-recombinant *E. coli* as the control. After the oral intake of sodium oxalate, the concentration of oxalate in urine increased exponentially for 3 hours. The oxalate concentration in urine was decreased significantly by *pBy* homogenates compared to control.

**Conclusions:**

We constructed the recombinant *E. coli* expressing *YvrK* gene and purified the recombinant oxalate decarboxylase successfully. Purified recombinant oxalate decarboxylase, as well as recombinant *E. coli* named *pBy*, showed the oxalate-degrading activity in *in vitro* and *in vivo* model.

## Background

Studies of the pathophysiology of urinary stones have focused on oxalate, since the concentration of urinary oxalate is more influential to the formation of calcium oxalate urolithiasis than is urinary calcium concentration [1]. Oxalate is the end product of a metabolic process; the compound is excreted into the urine. The majority of urinary oxalate is derived from the endogenous metabolism of glycine, glyoxylate, and ascorbic acid, and 40-50% is derived from oral ingestion [2]. Hyperoxaluria is induced by the increased production of oxalate through an abnormal endogenous metabolism or by elevated intestinal absorption of oxalate [3, 4]. Practically, it is difficult to control the endogenous metabolism of oxalate compared to the intestinal absorption of oxalate and some studies have sought to reduce intestinal absorption of oxalate.

*Oxalobacter formigenes* is an oxalate degrading bacterium colonizing the gastrointestinal tract of vertebrates including humans. The bacterium has a symbiotic relationship with its host by regulating oxalic acid absorption in the intestine and oxalate levels in plasma and urine [5, 6]. Decreased intestinal colonization of *O. formigenes* has been reported in recurrent calcium oxalate stone formers and patients with enteric hyperoxaluria [7, 8]. The absence of *O. formigenes* has been proposed as a risk factor for urolithiasis. However, *O. formigenes* is difficult to culture and isolate, and has complex pathways of oxalate degradation.

Recently, the entire DNA sequence of *Bacillus subtilis (B. subtilis)* has been determined, which revealed the existence of a gene designated *YvrK* gene that encodes a 43 KD-sized oxalate decarboxylase (OXDC). The OXDC degrades oxalate by a simple pathway [9, 10]. Previously we produced a *Yvrk* gene-recombinant *Escherichia coli (E. coli)* designated *pBy*, which expresses OXDC, and evaluated oxalate-degrading activity of the bacteria [11]. The aim of the present study was to purify the recombinant OXDC and evaluate the oxalate-degrading activity of purified OXDC, with the goal of developing a new treatment for hyperoxaluria in urinary stone patients.

## Methods

### Production of *Yvrk*-recombinant *E. coli*

The *Yvrk* gene-recombinant *E. coli* was produced as described previously [11]. *B. subtilis* strain 128 obtained from the Korean Gene Bank was cultured for 24 hours in Luria-Bertani (LB) broth (Difco, USA) in an aerobic environment of 95% O_2_/5% CO_2_ and temperature of 37°C. Cells were recovered by centrifugation at 5,000 × g at 4°C for 15 minutes. DNA was extracted from the cell pellet using a QIAamp DNA mini kit (Qiagen, USA).

The open reading frames and associated ribosome binding sites of *B. subtilis* 168 *YvrK* (GenBank™ accession no. 2832786) were amplified by polymerase chain reaction (PCR) using genomic DNA with the following oligonucleotides (shown 5′-3′): *Yvrk*-F, ATGAAAAAAC AAAATGACAT TCCG and *Yvrk*-R, TTTACTG CATTTCTTTT TCACTAC. The PCR products containing *YvrK* were digested with *NcoI* and *SalI.* The optimal reaction profile proved to be 94°C for 5 minutes, followed by 30 cycles of 94°C for 30 seconds for denaturation, 58°C for 30 seconds and 72°C for 30 seconds for annealing, and 72°C for 5 minute for primer extension. The PCR products were separated by gel electrophoresis in 2% agarose containing ethidium bromide, illuminated with ultraviolet light, and photographed for documentation.

The cloned *Yvrk* DNA segment was inserted into a pBAD/gIII-A vector containing a histidine tag. The DNA sequencing of the *Yvrk*-recombinant vector was done using an automated DNA sequencer (ABI Prism®, USA). The recombinant vector was transfected to non-pathogenic TOP 10 *E. coli* with a heat shock method involving 42°C for 90 seconds followed by 4°C for 10 minutes. The recombinant *E. coli* were cultured and selected in LB agar including ampicillin (100 μg/ml) at 37°C and 95% O_2_/5% CO_2_ for 24 hours.

*pBy* was grown at 37°C until an OD_600_ value of 0.5 with shaking. After heat-shocking at 42°C for 2 minutes [12], 5 μl/ml of serial dilution of L-arabinose (Invitrogen, USA; 0.02%, 0.2%, 2%, 20%) and 5 mM MnCl_2_ (Sigma-Aldrich, USA) were added. The cells were incubated at 37°C for 6 hours with shaking and harvested by centrifugation (13000 rpm, 10 minutes, 4°C) and stored at -80°C. Cells containing the empty vector were compared as negative control. The cell pellet was mixed with sodium dodecyl sulfate (SDS) sample buffer and visualized by 0.1% Brilliant Blue R (Sigma-Aldrich, USA) staining after separating by SDS-polyacrylamide gel electrophoresis (SDS-PAGE).

### Purification and characterization of recombinant enzyme

*pBy* cells were grown at 37°C until an OD_600_ value of 0.3 with shaking. After heat-shocking at 42°C for 2 minutes, 5 μl/ml of 2% L-arabinose and 5 mM MnCl_2_ were added. To increase the solubility of OXDC, the cells were incubated at 28°C for 24 hours and centrifuged. The cell pellet was stored at -80°C.

For purification under native conditions the cells pellet was thawed for 15 minutes and suspended in lysis buffer (50 mM NaH_2_PO_4_, 300 mM NaCl, 10 mM imidazole, pH 8.0). Cells were incubated on ice for 30 minutes, after which lysozyme was added to 1 mg/ml. The solution was sonicated on ice and the cell lysate was centrifuged at 10,000 × g for 30 min at 4°C. Ni-NTA Superflow (Qiagen, USA) was added to cleared lysate and mixed gently by shaking (200 rpm on a rotary shaker) at 4°C for 60 minutes. The lysate-Ni-NTA mixture was loaded into a column (BioRad, USA) and washed twice with wash buffer (50 mM NaH_2_PO_4_, 300 mM NaCl, 20 mM imidazole, pH 8.0), and the protein was eluted with elution buffer (50 mM NaH_2_PO_4_, 300 mM NaCl, 250 mM imidazole, pH 8.0). The cell lysate, wash and elutes were mixed with SDS sample buffer and evaluated by SDS-PAGE.

To determine the optimal pH and temperature, purified OXDC or PBS in pH 5, pH 6, or pH 7 was incubated with 5 mM MnCl_2_ and 1.5 mM sodium oxalate for 24 hours at 10°C, 20°C, 28°C, 37°C, 42°C, 60°C, or 70°C in atmospheric O_2_. The oxalate-degrading activity of purified OXDC protein was measured using an oxalate kit (Trinity Biotech, USA).

### *In vivo*oxalate-degrading activity

This study was approved by the Institutional Animal Care and Use Committee at Seoul National University Hospital Biomedical Research Institute. A transient hyperoxaluric rat model was established using 8-week-old Sprague–Dawley male rats [13]. Rats were anesthetized with an intramuscular injection of an 8:2 mixture of ketamine (Yuhan, Korea) and rompun (Bayer, Germany). A midline laparotomy was performed and a p10 tube was inserted into the bladder. Urine was collected hourly through the p10 tube before and until 3 hours after oral oxalate intake together with homogenates of *pBy* and TOP 10 *E. coli* (control). For oral oxalate intake, 0.5 ml of 1 mM sodium oxalate solution and 0.5 ml of *pBy* homogenates was administered through gastric gavage. Oxalate concentration in the hourly-collected urine was measured by a commercial oxalate kit.

## Results and discussion

### Production of *Yvrk*-recombinant *E. coli*

**Figure 1:**
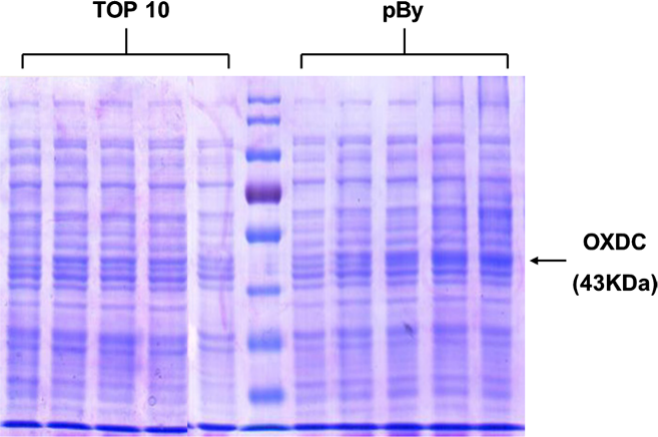
**Expression level of OXDC after incubation of** ***E. coli*** **TOP 10 and** ***pBy*** **cells with L-arabinose and MnCl**_**2**_ **.** The cloned DNA encoding OXDC was inserted into the pBAD/gIII-A vector and the plasmid vector transformed into *E. coli* TOP 10. Expression of OXDC was induced by different concentration of L-arabinose (0%, 0.02%, 0.2%, 2%, 20%) and MnCl_2_ and was confirmed using SDS-PAGE followed by staining with 0.1% Brilliant Blue R.

**Figure 2:**
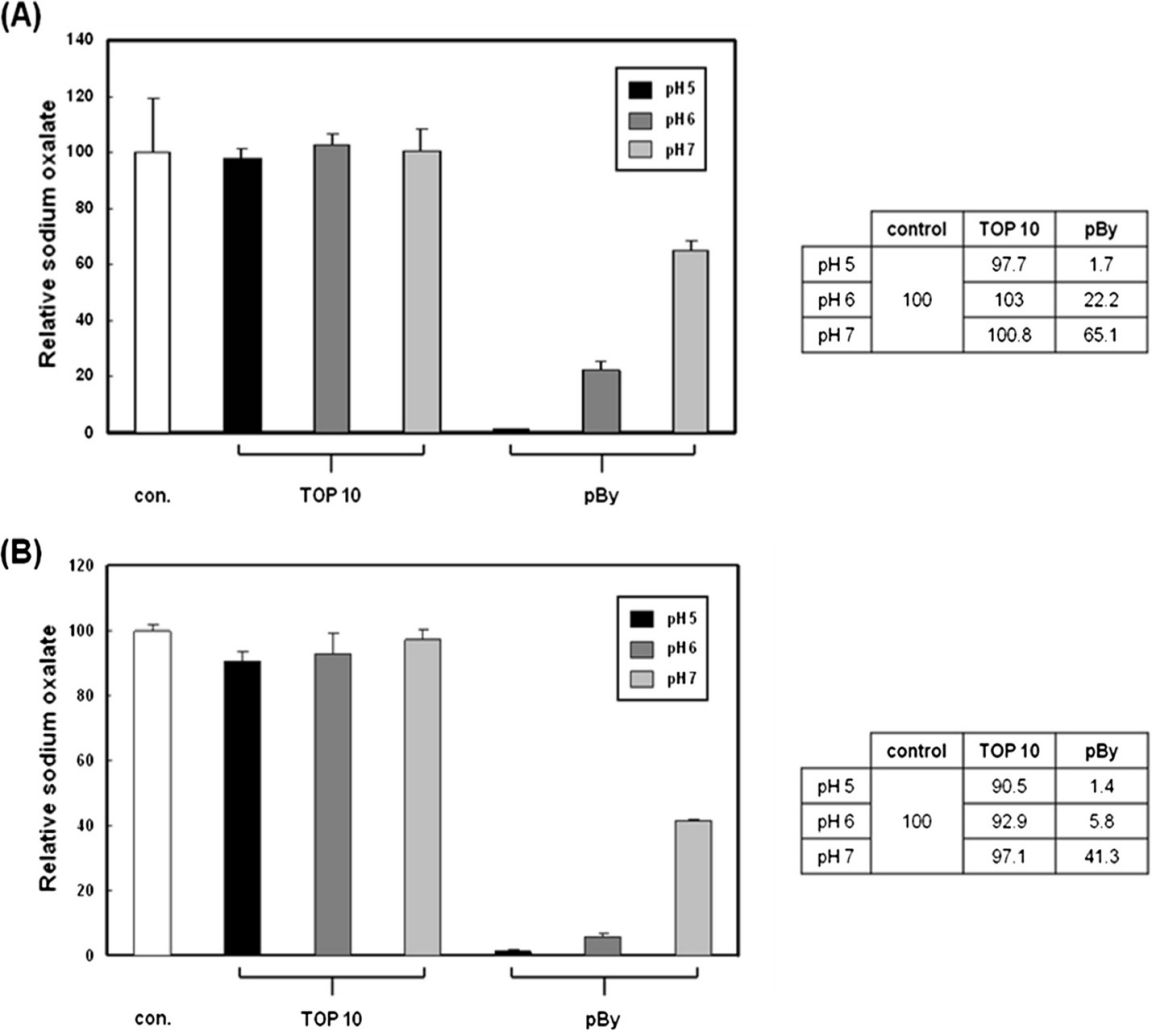
**Measurement of enzyme activity in** ***E. coli*** **TOP 10 and** ***pBy*** **cells at pH 5, pH 6 or pH 7.** *E. coli* TOP 10 and *pBy* cells were incubated with L-arabinose, MnCl_2_, and sodium oxalate at pH 5, pH 6, or pH 7 and the oxalate-degrading activity of OXDC in the supernatants was determined by an oxalate kit. Cells were incubated for 16 hours **(A)** and 24 hours **(B)**.

As we previously reported [11], 1.2 kb sized *Yvrk* gene was cloned from genomic DNA of *B. subtilis* by PCR. The *Yvrk* gene DNA were inserted into the pBAD/gIII-A vector. Automated DNA sequencing identified the same DNA sequence of *Yvrk* gene-recombinant pBAD/gIII-A vector to that of the *Yvrk* gene registered in National Center for Biotechnology Information. Figure 1 showed that the expression of OXDC was induced by treatment with L-arabinose in a dose-dependent manner. *E. coli* TOP 10 or *pBy* cells were grown in pH 5, pH 6 or pH 7 LB medium. When the cultures reached OD_600_ of 0.05, they were heat-shocked and incubated with L-arabinose, MnCl_2_ and sodium oxalate for 16 hours or 24 hours. LB medium was also incubated and used as control. The oxalate-degrading activity of *pBy* was determined by measurement of remaining oxalate. After incubation, amount of oxalate was not changed in *E. coli* TOP 10 at pH 5, 6 or 7 (Figure 2A). However, *pBy* showed oxalate-degrading activity which was dependent on pH and high in acidic condition (Figure 2B).

### Purification and characterization of recombinant enzyme

**Figure 3:**
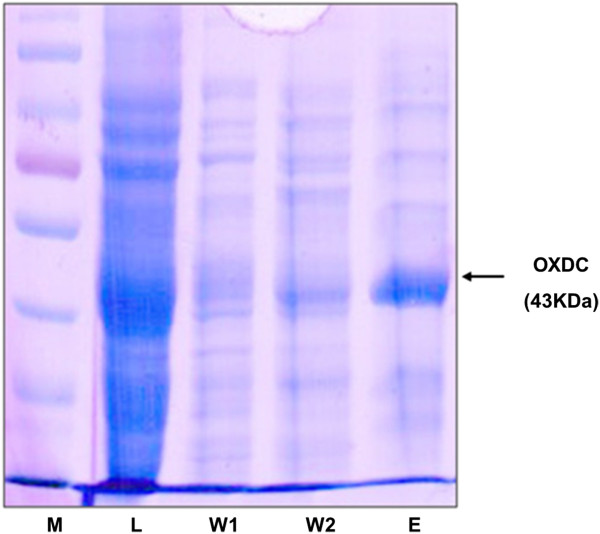
**Purification of recombinant OXDC by affinity chromatography and evaluation of the purity of recombinant proteins by SDS-PAGE.** The *pBy* cells were incubated with L-arabinose and MnCl_2_. After 6 hours of incubation, cells were harvested and purified using the Ni–NTA Superflow technique. The purity of recombinant proteins was determined by SDS-PAGE. M: markers; L: lysates; W1 & W2: first and second wash; E: elutes.

**Figure 4:**
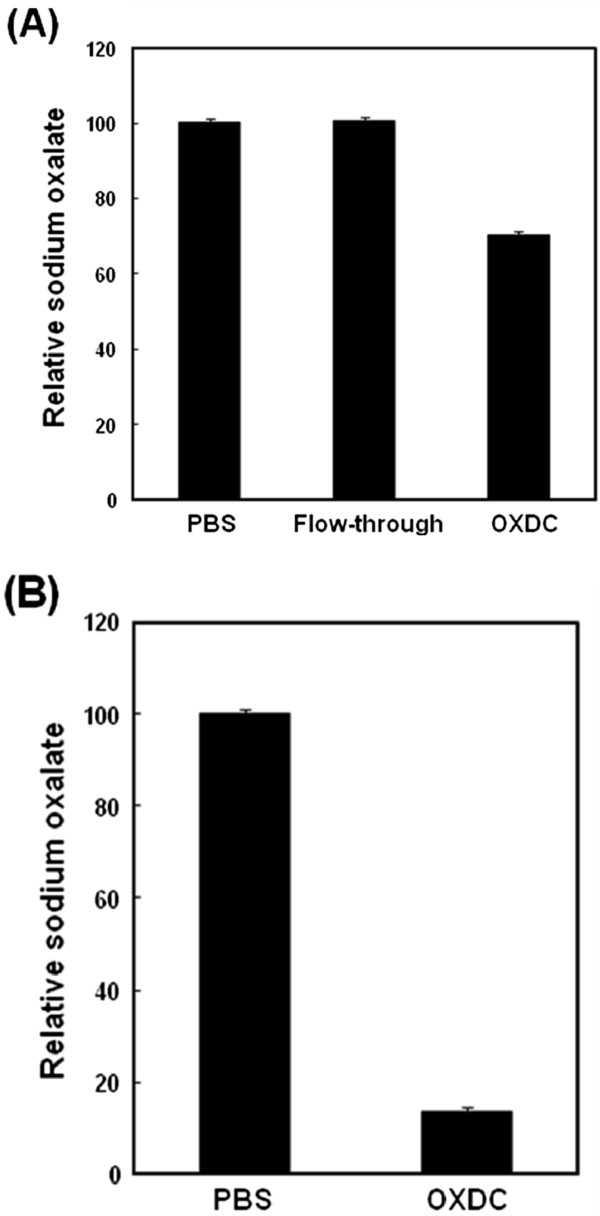
**Measurement of the oxalate-degrading activity of purified OXDC.** Expression of OXDC was induced by the addition of L-arabinose and MnCl_2_. After purification, the purified OXDC was incubated with sodium oxalate at room temperature and then enzymatic activity of purified OXDC was determined by oxalate kit. **(A)** The purified OXDC was incubated with sodium oxalate for 5 minutes. **(B)** The purified OXDC was incubated with sodium oxalate for 24 hours.

**Figure 5:**
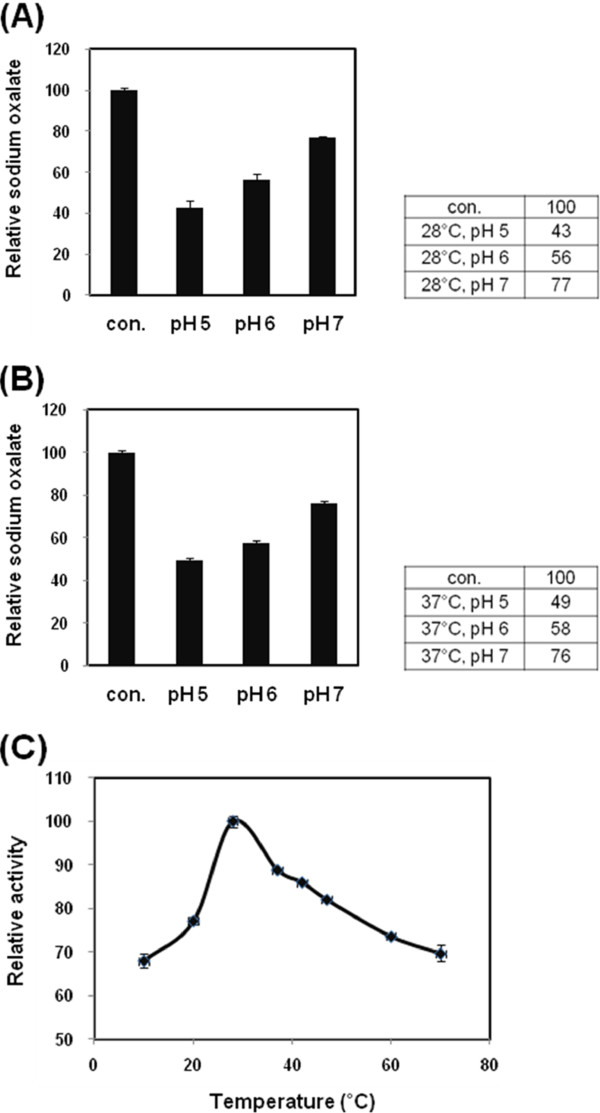
**Effect of pH and temperature on the oxalate-degrading activity.** Purified OXDC was incubated with oxalate at pH 5, 6 or 7 and enzyme activity of purified OXDC was determined by oxalate kit to investigate the optimal pH and temperature. Enzyme activity of purified OXDC (pH 5, pH 6 or pH 7) was measured at 28°C **(A)** or 37°C **(B)**. To investigate the optimal temperature, enzyme activity of purified OXDC at pH 5 was determined at 10°C, 20°C, 28°C, 37°C, 42°C, 60°C, or 70°C **(C)**.

The protein obtained as the purified enzyme was analyzed by SDS-PAGE. The molecular mass was 43 kDa (Figure 3). We found trace amounts of additional protein bands in this lane that might be partly host proteins and partly OXDC degradation products. To measure the activity of purified enzyme, purified OXDC or PBS at pH 5 was incubated with MnCl_2_ and sodium oxalate for 5 minutes or 24 hours at room temperature in atmospheric O_2_. After incubation, oxalate-degrading activity of enzyme was measured. As shown in Figure 4A, oxalate was slightly degraded by OXDC 5 minutes later. After incubation for 24 hours, significantly reduced oxalate was evident (Figure 4B). To characterize the OXDC, specific activity of the purified protein from *pBy* was measured at pH 5, 6 or 7 and various temperatures after incubation with MnCl_2_ and sodium oxalate for 24 hours. The optimal pH of OXDC was determined over the pH range of 5 to 7, in pH 1 intervals. At pH 5, the oxalate-degrading activity was the highest at 28°C and 37°C and the more it was incubated in acidic LB medium, the higher enzyme activity was (Figure 5A, B). At other temperatures, measurement of enzyme activity at pH 5-7 also was found similar results (data not shown). The optimal temperature of OXDC was investigated over the temperature range 10°C to 70°C. The highest activities at pH 5 (Figure 5C), pH 6 and pH 7 (data not shown) were at 28°C.

### *In vivo*oxalate-degrading activity

**Figure 6:**
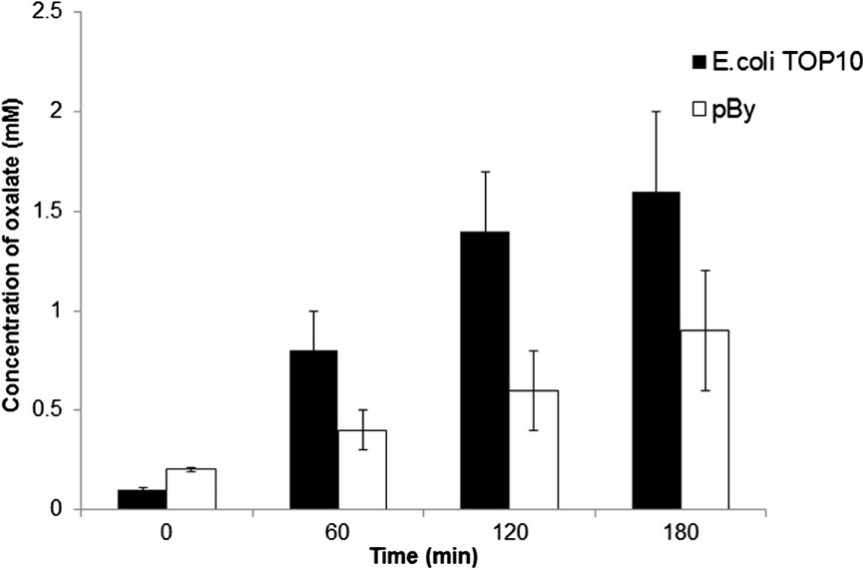
***In vivo*** **functional assay of** ***pBy*** **homogenate using hyperoxaluric rat model.** The oral intake of *pBy* homogenate reduced hyperoxaluria compared to control (*E coli* TOP 10).

*pBy* homogenates reduced urinary oxalate concentration significantly compared to *E.coli* TOP 10 in the transient hyperoxaluric rat model. The urinary oxalate concentration increased linearly at 1, 2, and 3 hours after gastric loading of 1 mM of sodium oxalate into rats (Figure 6).

## Discussion

Several human diseases have been linked to the excessive excretion of urinary oxalate. Mild forms of hyperoxaluria can lead to kidney stones. Kidney stone formation is a common urological disorder in the U.S. of nearly 13% in men and 7% in women. Recurrence of stone formation is common [14]. Larger amounts of oxalate in the urine can be caused by over-absorption of oxalic acid due to intestinal diseases. *O. formigenes*, degrades oxalate and maintains an important symbiotic relationship with its hosts by regulating oxalic acid absorption in the intestine [5].

*B. subtilis* is a Gram-positive, bacterium commonly found in soil. *B. subtilis* is rod-shaped endospore forming bacterium, which can tolerate extreme environment. A whole genome analysis of *B. subtilis* has revealed approximately 4,100 genes. Of these, 192 are indispensable and 79 are essential. *B. subtilis* harbors four bicupin-encoding genes: *YvrK*, *YoaN*, *YxaG* and *YwfC. YvrK* and *YoaN* encode OXDC and *YxaG* encodes quercetin dioxygenase [10]. The *Yvrk* gene has been identified in *B. subtilis* [9] and the produced OXDC degrades oxalate in a simple pathway.

However, *B. subtilis* does not express the *Yvrk* gene during growth in normal conditions and does not use oxalate as a metabolic source, unlike *O. formigenes,* which degrades oxalate by a metabolism to maintain its life. *B. subtilis* expresses *Yvrk* in the acidic condition when it forms endospores. Therefore, we did not manage *B. subtilis* directly in the experiment to control oxalate and produced *Yvrk*-recombinant *E. coli* (*pBy*) capable of degrading oxalate [11]. Our results show that *YvrK* gene from *B. subtilis* was successfully overexpressed in *E. coli* when *pBy* was incubated in the presence of manganese and oxygen, and the purified recombinant OXDC, as well as recombinant *E. coli* named *pBy*, had the oxalate-degrading activity. These results suggest that recombinant OXDC may be used for the treatment of human hyperoxaluria and prevent calcium oxalate nephrocalcinosis and urolithiasis.

The oxalate-degrading activity of *pBy* and recombinant OXDC in acidic conditions may be limited clinically because the main oxalate absorbing regions in humans are known the ileum and colon, whose pH is about 7. In this study, the capacity of *pBy* and the purified recombinant OXDC to degrade oxalate was reduced at near-neutral. However, some studies reported that stomach is also one of main places for oxalate absorption to be absorbed in human above ileum and colon [15–17]. One study evaluated six adult patients on permanent gastric tube feeding for various reasons, and reported a linear increase in the urinary oxalate excretion with increased gastric loading time [17]. The authors concluded that the stomach is not only just another oxalate absorption site but seems to be the critical site for intestinal oxalate absorption in an intact gastrointestinal tract.

We developed a transient hyperoxaluric rat model duplicating the performance of this prior study. Since we use rat models, we collected urine using a suprapubic catheter. The urinary oxalate concentration increased linearly at 1, 2, and 3 hours after gastric loading of oxalate into rats, similar to the previous findings with human patients [17]. When *pBy* homogenate was fed together with oxalate loading, the urinary oxalate concentration decreased compared to control. The results indicate that *pBy* homogenate acts in the stomach of rats and degrades oxalate *in vivo*. Although using *pBy* homogenate for *in vivo* functional assay is acceptable for providing preliminary evidence, it is probably more preferred to use purified enzyme, since purified enzyme will be free from contaminants and antigens of host cells, which otherwise may be present in the crude extract. Our next plan is to reveal that purified recombinant OXDC can reduce urinary oxalate concentration and inhibit stone formation in stone-forming rat models.

## Conclusions

In conclusion, we constructed a recombinant *E. coli* expressing the *YvrK* gene and purified the recombinant OXDC successfully. The purified enzyme, as well as the recombinant *E. coli* named *pBy*, showed the oxalate-degrading activity in *in vitro* and *in vivo* model. These data suggest that recombinant OXDC will provide a solution in the treatment of calcium oxalate stone and hyperoxaluria, which presently lack treatment modalities.

## Abbreviations

OXDC: Oxalate decarboxylase.
